# Associations between sedentary behaviour and physical activity in children and adolescents: a meta-analysis

**DOI:** 10.1111/obr.12188

**Published:** 2014-05-20

**Authors:** N Pearson, R E Braithwaite, S J H Biddle, E M F van Sluijs, A J Atkin

**Affiliations:** 1 School of Sport, Exercise & Health Sciences, Loughborough UniversityLoughborough, UK; 2 Department of Kinesiology and Recreation Administration, Humboldt State UniversityArcata, CA, USA; 3 The NIHR Leicester-Loughborough Diet, Lifestyle and Physical Activity Biomedical Research UnitLoughborough, UK; 4 MRC Epidemiology Unit & UKCRC Centre for Diet and Activity Research (CEDAR), University of Cambridge School of Clinical MedicineCambridge, UK

**Keywords:** Children, physical activity, sedentary behaviour

## Abstract

Physical activity and sedentary behaviour are associated with metabolic and mental health during childhood and adolescence. Understanding the inter-relationships between these behaviours will help to inform intervention design. This systematic review and meta-analysis synthesized evidence from observational studies describing the association between sedentary behaviour and physical activity in young people (<18 years). English-language publications up to August 2013 were located through electronic and manual searches. Included studies presented statistical associations between at least one measure of sedentary behaviour and one measure of physical activity. One hundred sixty-three papers were included in the meta-analysis, from which data on 254 independent samples was extracted. In the summary meta-analytic model (*k* = 230), a small, but significant, negative association between sedentary behaviour and physical activity was observed (*r* = −0.108, 95% confidence interval [CI] = −0.128, −0.087). In moderator analyses, studies that recruited smaller samples (*n* < 100, *r* = −0.193, 95% CI = −0.276, −0.109) employed objective methods of measurement (objectively measured physical activity; *r* = −0.233, 95% CI = −0.330, −0.137) or were assessed to be of higher methodological quality (*r* = −0.176, 95% CI = −0.215, −0.138) reported stronger associations, although effect sizes remained small. The association between sedentary behaviour and physical activity in young people is negative, but small, suggesting that these behaviours do not directly displace one another.

## Introduction

Sedentary behaviour has been the subject of increasing attention in recent years, from both the academic community and the popular press 1–5. It is defined as sitting and lying during waking hours when there is very low energy expenditure 6. Typical work or school-related sedentary behaviours include working at a desk or travelling by car, whereas leisure-time behaviours might involve TV viewing or recreational computer use. While all societies have seated behaviours, it is thought that contemporary lifestyles in developed countries involve very large amounts of sitting and that this has increased over the past decades 7. Much of this is attributed to new technologies 8,9.

Recent literature reviews in adults indicate that those adopting higher amounts of sitting relative to their counterparts have increased risk of non-communicable disease 10–12. Moreover, this is often shown to be somewhat independent of how much moderate-to-vigorous physical activity (MVPA) has been undertaken. Emerging evidence also indicates a potentially adverse impact of sedentary behaviour on health in children and adolescents 13,14. However, the effects are generally small for this largely healthy population 15,16 and current evidence is less convincing than for the effects of MVPA 17.

Until recent definitions provided some conceptual clarity, it was common to refer to low levels of physical activity as ‘sedentary’. It is important that a clear distinction between these clusters of behaviours is made. This is pertinent because evidence indicates that even those meeting guidelines for physical activity may still accumulate considerable sedentary time. One of the first studies to show this was Marshall and colleagues' cluster analysis of adolescents from the UK and the USA 18. In both boys and girls, clusters of participants were identified that reported higher than average levels of physical activity along with elevated levels of screen-based sedentary behaviour or sedentary socializing activities.

Alongside this literature, it has been suggested that sedentary behaviours may hinder participation in physical activity. This is sometimes referred to as the ‘displacement hypothesis’, whereby one behaviour (sitting) displaces another (physical activity) 19. While displacement may occur at a single point in time (we cannot do both behaviours simultaneously), it may not be true across the day or week. In their seminal review of factors associated with physical activity (correlates) in young people, Sallis *et al*. showed that it was not total sedentary time that was associated with lower levels of physical activity but rather sedentary behaviour after school and at weekends 20.

In addition to the temporal context of sedentary behaviour, the way we assess behaviours may also be important. Objective assessments of sedentary time typically involve a movement or leg-angle sensor that estimates time sitting 21. However, without additional information, one cannot determine what behaviours are being undertaken. Self-report methods, such as questionnaires, can be most helpful in identifying what people are doing, although the precision of time estimates is often low 21. To answer the question whether time in sedentary behaviour replaces time that might otherwise be spent in active pursuits, it might be wise to assess both total time and types of behaviour across the day. This will necessitate the use of both objective and self-report methods.

Given that energy expenditure and postural allocation during the waking day can vary across the spectrum of sedentary to vigorous activities, it is important that we better understand the associations between these groups of behaviours. The aim of the current study was to systematically review and meta-analyse peer-reviewed research describing the association between sedentary behaviour and physical activity in children and adolescents.

## Methods

This study followed the procedures for a meta-analysis as documented in the PRISMA statement 22.

### Search strategy

Search strategies were built around two groups of keywords: population (e.g. ‘girls’, ‘boys’, ‘children’, ‘adolescents’, ‘pre-school’, ‘youth’, ‘teenagers’) and behaviour (e.g. ‘sedentary behaviour’, ‘television viewing’, ‘sitting’, ‘physical activity’, ‘activities’, ‘exercise’). ScienceDirect, PubMed, PsycINFO, Web of Science, Cochrane Libraries and EPPI Centre databases were searched using the key terms up to and including August 2013. In addition, manual searches of personal files were conducted along with screening of reference lists of previous sedentary behaviour and/or physical activity reviews 14,16,20,23–34 and identified articles.

### Inclusion and exclusion criteria

For inclusion, studies were required to (i) be an observational study or baseline of an intervention study; (ii) include children aged ≤11 years and/or adolescents aged 12–18 years (or a mean within these ranges) as participants of the study; (iii) include at least one quantitative assessment of sedentary behaviour and at least one quantitative assessment of physical activity behaviour; (iv) measure the association between at least one domain of sedentary behaviour and one domain of physical activity; and (v) be published in the English language.

### Identification of relevant studies

**Figure 1:**
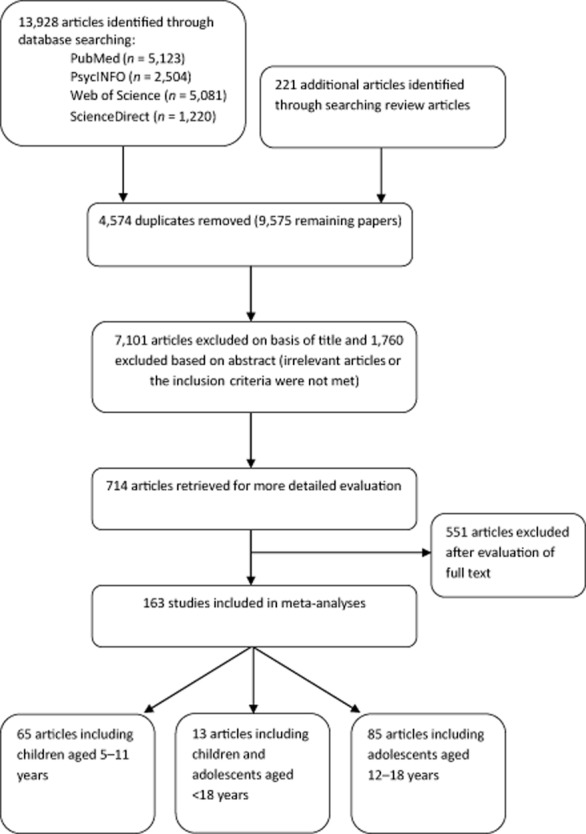
Flow of information through different phases of the meta-analyses.

Potentially relevant articles were examined by two authors (NP and AA) who (i) screened the titles; (ii) screened the abstracts; and (iii) if abstracts were not available or did not provide sufficient data, retrieved the entire article which was then screened to determine whether it met the inclusion criteria (see Fig. 1). At each stage, a selection of papers was cross-checked by a third author (SJHB). Where there was uncertainty or disagreement regarding inclusion, a discussion was held between the three authors to reach a decision.

### Data extraction and coding

Detailed information was extracted from each article by two authors (NP and AA) and included sample characteristics (sample size, age, gender, socio-economic status and ethnicity), country of study, study design, domain of sedentary behaviour assessed (e.g. screen time), domain of physical activity assessed (e.g. moderate physical activity), measures used for sedentary behaviour and physical activity (e.g. subjective or objective), temporal dimension of physical activity or sedentary behaviour assessment (e.g. leisure time or weekday only) and information on study quality (described below). Where studies combined multiple sources of assessment to derive a marker of sedentary behaviour that reflected multiple domains or the specific derivation of the sedentary behaviour construct was unclear, we refer to ‘composite sedentary behaviour’ (*k* = 48). Information about the association between sedentary behaviour and physical activity, including the data format for the association (e.g. correlation or odds ratio) and the reported statistical data, were extracted by NP and AA for use in calculating effect sizes. Data were extracted using a standard data extraction instrument developed specifically for this study. When key information was missing from an article, three attempts were made to reach the corresponding author via e-mail before using the available data or eliminating the study from the analysis.

### Study quality

A scale assessing methodological quality was developed, informed by previously reported checklists 31,33,34. The instrument included items related to the quality of reporting (three items, with an additional item for prospective studies) and study quality (validity/precision: eight items with one additional item for prospective studies) and all included studies were assessed against the scale by two reviewers. The 11-item instrument (13 items for prospective studies) is available from the authors. Items were marked ‘positive’ (scored 1), ‘negative’ (scored 0) or ‘not sufficiently described’ (scored 0). Total scores for quality of reporting and for study quality were calculated by adding all positive scores for each assessed study. The scoring system placed an emphasis on positive scores. Negative and not sufficiently described items were treated equally; no points were scored for either. For analytical purposes, study quality scores (ranging from 0 to 8 for cross-sectional studies and from 0 to 9 for prospective studies) were categorized into ‘high quality’ (scores of 5 or more) and ‘low quality’ (scores of 0–4) and used as a potential moderator.

### Outliers and publication bias

Data were screened to identify outliers and determine the potential influence of publication bias. Studies with relative residual scores outside the 95 percentile of the mean effect size (*z*-score ≥ ± 1.96) were deemed to be outliers. Sensitivity analyses were conducted to examine the impact of retention/removal of outliers on the overall effect estimate (‘one study removed’ procedure) 35. *A priori* it was determined that studies should be retained when upon removal the overall effect size remained significant and within the 95% confidence interval. Evidence of publication bias was assessed in three ways: inspection of funnel plots 36, application of the ‘trim and fill’ procedure 37,38 and calculation of a ‘fail-safe *N’* estimate 39.

### Effect size calculations

Random effects meta-analysis was used to derive a pooled estimate of the association between sedentary behaviour and physical activity. Studies that calculated correlation coefficients or those that treated physical activity as the outcome were synthesized in the main analysis. Studies that examined associations between sedentary behaviour and *low* physical activity (e.g. physical activity outcome: 0 = high activity, 1 = low activity) and those that treated sedentary behaviour as the outcome were analysed separately. Fisher's *z* transformation was applied to correlation coefficients to permit the calculation of the relevant statistics (variance, standard error, confidence intervals) before converting back to the correlation to report the summary effect size. An inverse variance weighting procedure for independent effect sizes was used to improve overall precision, as recommended when several metrics are utilized to compute a combined estimate of the total effect 40,41. An independent sample (*k*) was used as the unit of analysis. Pearson's *r* was the effect size metric selected to report results. Cohen's criteria for small (>0.20), moderate (>0.50) and large (>0.80) effect sizes was used to aid the interpretation of results 42.

Between-study heterogeneity was quantified using the *Q*-value, tau-squared (τ^2^), and *I*-squared (*I*^2^) statistics. Moderator analyses were conducted to examine associations between physical activity and sedentary behaviour sub-domains (e.g. TV viewing) and the influence of selected demographic and methodological characteristics, including age group and the use of subjective vs objective methods of assessment. Analyses were performed using Comprehensive Meta-Analysis (version-2) 43.

## Results

Computerized and manual searches produced 14,149 ‘hits’, of which 163 studies met the inclusion criteria (Fig. 1). From the included studies, data were extracted for 254 independent subsamples. Included studies were published between 1987 and 2013 and most were conducted in the USA (*n* = 65) or Europe (*n* = 39). Subsamples mostly described cross-sectional associations (*k* = 212) based upon self- or proxy reports of physical activity (*k* = 179) or sedentary behaviour (*k* = 209). Descriptive characteristics and references of studies that included children only (≤11 years, *n* = 65), children and adolescents (*n* = 13), and adolescents only (12–18 years, *n* = 85) are presented in Supporting Information Tables S1–S3.

### Associations between physical activity and sedentary behaviour

The associations of overall sedentary behaviour and sedentary behaviour sub-domains with physical activity are presented in Table 1. In the summary meta-analytic model (*k* = 230), a significant, but small, negative association between physical activity and sedentary behaviour was observed. When examined separately, small negative associations were also observed for Internet use, screen time (combined TV viewing, computer use, video game play), composite sedentary behaviour and TV viewing. There was evidence of substantial heterogeneity between studies, as reflected in the significant *Q*-values and high *I*^2^ statistics, which exceeded 90% in most cases.

**Table 1:** Associations of overall and sub-domains of sedentary behaviour with all physical activity outcomes (subsample is the unit of analysis)

	Effect size statistics†						Heterogeneity statistics			Publication bias
	*k*	*r*	SE	*s*^2^	95% CI	*Z*	*Q*	τ^2^	*I*^2^	Fail safe *N*
Total	230	−0.108	0.011	0.000	−0.128, −0.087	−10.23***	13,072.80***	0.022	98.25	18,025
Computer	37	−0.018	0.010	0.000	−0.038, 0.001	−1.84	826.17***	0.003	95.64	78
Homework	5	0.014	0.015	0.000	−0.043, 0.095	−0.93	2.59	0.000	0.00	0
Internet	3	−0.051	0.023	0.001	−0.097, −0.006	−2.20*	0.745	0.000	0.000	0
Reading	4	−0.009	0.015	0.000	−0.039, 0.021	−0.59	5.69	0.000	47.32	0
Screen time	85	−0.080	0.010	0.000	−0.101, −0.060	−7.68***	2,395.14***	0.006	96.49	9,071
Television	101	−0.064	0.010	0.000	−0.084, −0.045	−6.53***	3,059.04***	0.007	96.73	6,004
Video games	26	−0.002	0.021	0.000	−0.043, 0.040	−0.07	2,250.12***	0.009	98.89	1,009
Composite sedentary behaviour	48	−0.265	0.051	0.003	−0.364, −0.165	−5.22***	6,936.57***	0.119	99.32	2,904

^*^P < 0.05; ^*^^*^^*^P < 0.001.

†Fisher's *Z* was used to calculate effect size statistics; *k*, number of effect sizes; *r*, effect size; SE, standard error; *s*^2^, variance; 95% confidence interval; *Z*, test of null hypothesis; *Q*, total *Q*-value used to determine heterogeneity; τ^2^, between study variance in random effects model; *I*^2^, the percentage of total variation across studies that is due to heterogeneity rather than chance. Fail safe *N*: the potential for publication bias to have influenced the results of a meta-analysis. Fail safe *N* is the number of additional studies (studies in which the effect was zero) that would be needed to increase the *P* value for the meta-analysis to above 0.05.

### Outliers and publication bias

Outlier analysis identified 14 studies with large residual values (*z*-score ≥ ± 1.96) 17,18,44–55. All studies were retained in final models as results from the ‘one study removed’ procedure revealed marginal impact on the overall effect estimate, which remained significant and within the 95% confidence interval. Evidence of publication bias was minimal, as review of funnel plots indicated a symmetrical distribution, the ‘trim and fill’ procedure did not add studies to the funnel plot, and the ‘fail safe *N*’ calculations indicated that a large number (>1,000 in most cases) of additional non-significant studies were needed to nullify the results.

### Moderator analyses

Subgroup analyses for sample and study characteristics are presented in Tables 2 and 3, respectively. For sample characteristics, sample size was the only category to have a significant difference between groups, with studies based upon smaller samples producing larger effect sizes for the association between sedentary behaviour and physical activity. Heterogeneity statistics indicated that the sample grouping within categories had smaller variance (tau-squared) coefficients; however, there was inconsistency (large *I*^2^) between studies. With regard to study-level characteristics, significant differences between groups were observed for the type of physical activity and sedentary behaviour assessment used. In both cases, stronger associations were observed in studies that used objective methods of measurement. A significant difference by study quality was also observed, such that higher quality studies produced stronger negative associations. Analysis of the heterogeneity statistics indicated variability and inconsistent findings within category sub-groupings (large *Q* and *I*^2^ values).

**Table 2:** Sample characteristics subgroup analysis for the association between sedentary behaviour (all variables) and physical activity (all variables) (subsample is the unit of analysis)

Sample characteristics	Effect size statistics†						Heterogeneity statistics		
	*k*	*r*	SE	*s*^2^	95% CI	*Z*	*Q*	τ^2^	*I*^2^
Age group							6.53^B^		
Children 0–5	19	−0.053	0.026	0.001	−0.104, −0.001	−1.99*	72.99***	0.008	75.34
Children 5–11	81	−0.138	0.027	0.001	−0.190, −0.086	−5.19***	3,737.31***	0.050	97.86
Adolescents 12–18	14	−0.066	0.031	0.001	−0.127, −0.005	−2.12*	137.57***	0.011	90.55
Adolescents 12–15	92	−0.089	0.009	0.000	−0.108, −0.072	−9.73***	2,465.36***	0.006	96.31
Adolescents 16–18	7	−0.032	0.015	0.000	−0.062, −0.002	−2.09*	15.84*	0.001	62.12
Children and adolescents	17	−0.121	0.080	0.006	−0.277, −0.036	−1.51	36.38***	0.105	99.56
Gender							3.65^B^		
Boy only	51	−0.120	0.018	0.000	−0.155, −0.085	−6.66***	1,346.87***	0.012	96.29
Girls only	61	−0.088	0.011	0.000	−0.110, −0.066	−7.82***	623.09***	0.005	90.37
Boys and girls	118	−0.108	0.023	0.001	−0.153, −0.063	−4.74***	10,233.59***	0.058	98.85
Sample size							13.09^B^**		
<100	28	−0.193	0.043	0.002	−0.276, −0.109	−4.52***	66.51***	0.028	59.41
101–500	63	−0.148	0.035	0.001	−0.217, −0.079	−4.22***	1,386.89***	0.073	95.53
501–1,000	23	−0.065	0.019	0.000	−0.102, −0.027	−3.39***	136.80***	0.070	83.92
>1,000	116	−0.085	0.014	0.000	−0.113, −0.058	−6.20***	11,385.38***	0.020	98.99
Country							2.89^B^		
Australia/New Zealand	30	−0.114	0.028	0.001	−0.168, −0.060	−4.12***	522.12***	0.019	94.45
Europe	70	−0.130	0.023	0.001	−0.174, −0.085	−5.74***	4,162.47***	0.033	98.34
USA	97	−0.100	0.015	0.000	−0.129, −0.071	−6.79***	1,523.98***	0.016	93.70
Multiple	4	−0.136	0.083	0.007	−0.298, 0.027	−1.64***	6,374.26***	0.027	99.95
Other	29	−0.059	0.015	0.000	−0.094, −0.024	−3.27***	264.18***	0.007	89.40

^*^^*^^*^P < 0.001; ^*^^*^P < 0.01; ^*^P < 0.05.

†Fisher's *Z* was used to calculate effect size statistics; *k*, number of effect sizes; *r*, effect size; SE, standard error; *s*^2^, variance; 95% CI, 95% confidence interval; *Z*, test of null hypothesis; τ^2^, between study variance in random effects model; *I*^2^, total variance unexplained by moderator; B, between *Q*-value used to determine significance between subgroups.

**Table 3:** Study characteristics subgroup analysis for the association between sedentary behaviour (all variables) and physical activity (all variables) (subsample is the unit of analysis)

Study characteristics	Effect size statistics†						Heterogeneity statistics		
	*k*	*r*	SE	*s*^2^	95% CI	*Z*	*Q*	τ^2^	*I*^2^
Study type							1.59^B^		
Cross sectional	209	−0.113	0.111	0.000	−0.136, −0.091	−10.04***	12,802.08***	0.023	98.36
Prospective	21	−0.049	0.018	0.000	−0.084, −0.013	2.66***	160.19***	0.005	87.52
PA assessment							60.16^B^**		
Subjective	176	−0.067	0.006	0.000	−0.081, −0.057	−11.54***	2,549.65***	0.004	93.16
Objective	54	−0.233	0.049	0.002	−0.330, −0.137	−4.73***	5,168.62***	0.121	98.98
SB assessment							178.68^B^**		
Subjective	209	−0.071	0.006	0.000	−0.082, −0.060	12.35***	2,749.53***	0.005	92.44
Objective	21	−0.449	0.066	0.004	−0.578, −0.320	−6.81***	1,470.73***	0.085	98.64
Timing of PA measure							5.52^B^		
After-school	7	−0.061	0.059	0.003	−0.177, 0.055	−1.03	77.99***	0.019	92.31
Leisure time	37	−0.036	0.008	0.000	−0.053, −0.019	−4.25***	486.29***	0.001	92.60
Whole days	163	−0.129	0.019	0.000	−0.166, −0.092	−6.91***	11,303.29***	0.052	98.57
Weekdays	6	−0.083	0.025	0.001	−0.131, −0.035	−3.38*	7.34*	0.001	31.86
Weekends	6	−0.064	0.028	0.001	−0.118, −0.010	−2.31*	5.00	0.000	0.09
Not specified	11	−0.101	−0.057	0.003	−0.214, 0.012	−1.76	202.36***	0.033	95.06
Timing of SB measure							7.76^B^		
After-school	9	−0.094	0.023	0.001	−0.138, −0.049	−4.15***	36.80***	0.003	78.26
Leisure time	23	−0.045	0.010	0.000	−0.065, −0.025	−4.42***	373.73***	0.001	94.11
Whole days	163	−0.120	0.019	0.000	−0.158, −0.083	−6.32***	11,582.38***	0.054	98.60
Weekday/weekend	2	−0.062	0.055	0.003	−0.169, 0.046	−1.13	28.07***	0.006	96.42
Weekdays	18	−0.072	0.013	0.000	−0.098, −0.046	−5.39***	44.82***	0.001	62.07
Weekends	10	−0.087	0.068	0.005	−0.220, 0.045	−1.29	125.81***	0.035	92.85
Not specified	5	−0.141	0.110	0.012	−0.356, 0.073	−1.29	201.07***	0.058	98.01
Study quality							17.61^B^**		
Low	147	−0.064	0.008	0.000	−0.079, −0.048	−7.89***	1,265.99***	0.007	88.47
High	83	−0.176	0.020	0.000	−0.215, −0.138	−8.88***	11,550.32***	0.029	99.29

^*^^*^^*^P < 0.001; ^*^^*^P < 0.01; ^*^P < 0.05.

†Fisher's *Z* was used to calculate effect size statistics; *k*, number of effect sizes; *r*, effect size; SE, standard error; *s*^2^, variance; 95% CI, 95% confidence interval; *Z*, test of null hypothesis; τ^2^, between study variance in random effects model; *I*^2^, total variance unexplained by moderator; B, *Q*-value used to determine significance between subgroups.

PA, physical activity; SB, sedentary behaviour.

### Additional analyses

A small number of studies (*n* = 14; *k* = 22) performed analyses to examine the association between sedentary behaviour and low levels of physical activity. In meta-analytic models, the association was small but positive (*r* = 0.067, 95% CI = 0.033, 0.101), consistent with results from the main analysis. Four studies, reporting data from four independent samples, presented analyses in which sedentary behaviour was the outcome variable. Associations were small and negative (*r* = −0.019, 95% CI = −0.118, 0.080). We were unable to integrate one study into the meta-analysis due to the method of analysis employed 56. In this case, relative to those with high activity levels, boys with low activity levels were more likely to exceed 2 h d^–1^ of screen time and those with moderate activity levels were more likely to exceed 1 h d^–1^ of homework. In girls, those who reported low or moderate levels of activity were less likely to exceed 2  h d^–1^ of screen time but more likely to report greater than 1 h d^–1^ of homework, compared to the high active group.

## Discussion

In this systematic review and meta-analysis, we observed a significant, but small, negative association between sedentary behaviours and physical activity in children and adolescents. In moderator analyses, studies that recruited smaller samples, employed objective methods of measurement or were assessed to be of higher methodological quality reported stronger associations, although the magnitude of effect remained small or small to moderate 42. Findings provide little support for the ‘displacement hypothesis’, which asserts that engagement in sedentary behaviours may displace physical activity in young people 19. Overall, the association is generally weak and may therefore have limited relevance from a clinical or behavioural perspective.

Overall sedentary behaviour and specific sub-domains of sedentary behaviour were negatively associated with physical activity. The direction of the association is consistent with the displacement hypothesis, but the small magnitude does not support the existence of a direct substitution effect. Supportive of our findings, evidence from experimental research indicates that the association between sedentary behaviour and physical activity may be asymmetrical, such that modification of children's sedentary behaviour will impact upon physical activity only under particular conditions 57. It seems likely that in most children, sedentary and physically active behaviours may co-exist without detriment. Findings indicate that physical activity and sedentary behaviour should be considered distinct constructs, and assessments in one domain should not be applied as markers of the other 58–60. In developing interventions to promote physical activity, strategies targeting a reduction in sedentary behaviour may be beneficial only when employed as part of a broader package of measures targeting the determinants of physical activity. With regard to obesity prevention, both insufficient physical activity and excessive sedentary behaviour have been implicated as potential causes of obesity, but the evidence on this issue remains mixed and is characterized by numerous methodological limitations 61. Further research is required to clarify the relative and interacting impact of physical activity and sedentary behaviour on weight status in this population.

Small inverse associations were observed between specific sedentary behaviours, including Internet use, screen time and TV viewing, and physical activity. The information technology landscape has evolved rapidly in recent years, impacting significantly on the way that young people communicate and consume media content. While TV remains the most prominent leisure-time sedentary behaviour, it is increasingly being accessed through alternative platforms (mobile phones, tablet computers), which often are portable and multifunctional 62–65. Considered alongside the ‘active’ video games movement, it may no longer be appropriate to infer sedentariness 6 from reported behaviour, even those related to screen-use. This may, in part, account for the relatively weak associations observed in the current review. Moreover, there is increasing recognition of the pro-social aspects of media use in young people and the important role that it can play in education, for example 65. A multifaceted approach to the assessment of sedentary behaviour will enable a more nuanced understanding of children's sedentary behaviour patterns and how they interact with physical activity and development.

In moderator analyses, stronger inverse associations between physical activity and sedentary behaviour were observed in studies that employed objective methods of measurement. Even within these subgroups, however, correlations remained less than 0.5. One possible explanation for this finding is that accelerometry for example provides a measure of overall sedentary time rather than an assessment of specific sedentary behaviours. Relative to a single sedentary behaviour, aggregated sedentary time may correlate more strongly with physical activity because it accounts for a larger proportion of daily time use; this dependency may be further inflated when both constructs are measured using the same device. In addition, accelerometer counts per minute (sometimes used as a marker of overall physical activity intensity) and accelerometer assessed sedentary time are not wholly independent, which may have biased estimates of the association in the small number of studies that correlated these two constructs. Moderator effects observed for smaller sample size and higher methodological quality may also have been driven, at least in part, by the use of objective monitoring. Cost and feasibility constraints sometimes limit the use of objective measures in very large epidemiological studies, and our quality assessment tool included items related to the use of valid and reliable instruments to assess physical activity and sedentary behaviour.

No differential effect was observed when sedentary behaviour or physical activity assessments were restricted to specific times of the day or week. This was somewhat surprising as it may be expected that relative to the entire day or week, the allocation of time to one behaviour is more likely to displace time available to engage in alternative activities when focusing on a shorter time frame (e.g. after-school). One explanation for this finding is that some studies for example examined the association of sedentary behaviour during a specific period with overall physical activity, rather than examining the inter-relationship between behaviours assessed over the same reference period. It remains plausible, therefore, that within specific time frames, particularly those where children have relatively free choice, sedentary and physically active behaviours may compete for time. This may be a valuable avenue for future research examining the interplay between these groups of behaviours and the development of time-specific intervention strategies.

The strengths of this review include the use of meta-analysis to provide a quantitative synthesis of included studies and the exploration of differential associations across a broad range of sample and study characteristics. Numerous methods to assess publication bias were employed and none indicated that this was a major concern. We utilized broad search criteria, including both electronic and manual sources, and a large number of studies were screened for eligibility. However, few of the included studies were intended to address directly the question of interest to this review. The association between sedentary behaviour and physical activity was frequently reported as a descriptive characteristic within a methods, results or discussion section. Therefore, we cannot rule out the possibility that some relevant studies were not identified for the current synthesis. To facilitate the examination of study quality as a potential moderator, an arbitrary threshold was applied to distinguish low and high quality. We acknowledge the limitation of dichotomizing a continuous construct in this way. In addition, searches were confined to studies published in peer-reviewed journals and those written in English. Due to the large number of studies meeting inclusion criteria, we explored associations between sedentary behaviour and all physical activity outcomes combined. It is possible that differential associations may be observed across different physical activity domains (e.g. sports participation, active travel); however, we felt that stratified analysis of physical activity outcomes was beyond the scope of the current review.

## Conclusion

Despite the established health benefits, a substantial proportion of young people fail to meet public health guidelines for physical activity and participation declines during the transition from childhood to adolescence 66,67. Concurrently, sedentary behaviours, such as TV viewing and computer use, are highly prevalent in this population 62,68,69. The question of whether sedentary behaviours displace participation in physical activity, therefore, is highly relevant and of interest to a range of stakeholders, including behavioural scientists, policy makers and parents. Findings of the current review indicate that sedentary behaviour is inversely associated with physical activity in young people, but the relationship is weak, suggesting that these behaviours should not be considered functional opposites or ‘two sides of the same coin’. The complex interplay between sedentary and physically active behaviours, including their shared and unique determinants, should be considered in the development and evaluation of behaviour change interventions.

## Conflict of interest statement

The authors declare no conflicts of interest.

## Supporting Information

**Table S1.** Descriptive characteristics of studies including children aged 0–11 years.

**Table S2.** Descriptive characteristics of studies including children and adolescents.

**Table S3.** Descriptive characteristics of studies including adolescents aged 12–18 years.
