# Supplementary material for: Associations between sedentary behaviour and physical activity in children and adolescents: a meta-analysis
Source: Obes Rev. 2014 May 20;15(8):666–75. doi: 10.1111/obr.12188 (PMC4282352; doi:10.1111/obr.12188)
Supplement: Supplementary file 1 — Table S1. Descriptive characteristics of studies including children aged 0–11 years. [file obr0015-0666-sd1.docx]

**Supplementary Table 1.** Descriptive characteristics of studies including children aged 0-11 years

| **Author** | **Country** | **Age group** | **Gender** | **Sample size** | **Study design** | **Exposures / Outcomes** | |  | **Assessment method** | | **Quality rating** |
| --- | --- | --- | --- | --- | --- | --- | --- | --- | --- | --- | --- |
|  |  |  |  |  |  | **SB** | **PA** |  | **SB** | **PA** |  |
| **Adams et al. 2010**(1) | USA | 5-11 | G | 101-500 | C | ST | SP |  | PR | PR | L |
| **Anderson et al. 2009**(2) | USA | 5-11 | B & G | 501-1000 | C | C, TV | MVPA, SP |  | SR | SR | H |
| **Anderson et al. 2008**(3) | USA | 5-11 | B & G | 1000+ | C | ST | AP |  | PR | PR | L |
| **Bergh et al. 2011**(4) | Norway | 5-11 | BG | 1000+ | C | C | MVPA |  | SR | OB | H |
| **Brophy et al. 2011**(5) | UK | 511 | BG | 1000+ | C | TV | AT, EX |  | PR | PR | L |
| **Brown et al.**  **2010**(6) | Australia | 5-11 | BG | 1000+ | C, P | TV | MVPA |  | PR | PR | L |
| **Brown et al.**  **2011**(7) | Australia | 5-11 | BG | 1000+ | C | TV | PA |  | PR | PR | L |
| **Byun et al.**  **2011**(8) | USA | 0-5 | B & G | 101-500 | C | SB | SP |  | OB | PR | H |
| **Calderon et al. 1996**(9) | USA | 5-11 | G | <100 | C | ST | PA |  | SR | SR | L |
| **Caradente et al. 2009**(10) | Italy | 5-11 | BG | 1000+ | C | ST | OG, SP, PA |  | SR | SR | L |
| **Cox et al.**  **2012**(11) | Australia | 0-5 | BG | 101-500 | C | TV | LPA, MVPA |  | PR | PR | L |
| **Drenowatz et al. 2012**(12) | USA | 5-11 | BG | 101-500 | C | ST | PA |  | SR | SR | L |
| **Dumith et al. 2010†**(13) | Brazil | 5-11 | BG | 1000+ | C | ST | I |  | SR | SR | L |
| **Dumith et al. 2012**(14) | Brazil | 5-11 | B & G | 1000+ | P | ST | PA |  | SR | SR | L |
| **DuRant et al. 1994**(15) | USA | 0-5 | BG | 101-500 | C | TV | PA |  | OB | OB | L |
| **Ekstedt et al. 2013**(16) | Sweden | 5-11 | BG | 1000+ | C | SB | CPM, MVPA |  | OB | OB | H |
| **Esmaeilzadeh et al. 2013**(17) | Iran | 5-11 | B | 101-500 | C | ST | PA |  | PR | PR | L |
| **Foley et al.**  **2009**(18) | USA | 5-11 | BG | <100 | C | ST | PA |  | PR | OB | L |
| **Fulton et al. 2009**(19) | USA | 5-11 | BG | 101-500 | C | SB | MVPA |  | SR | SR | L |
| **Hager et al. 2006**(20) | USA | 5-11 | B & G | 101-500 | C | TV | PA |  | SR | OB | L |
| **Hands et al. 2011**(21) | Australia | 5-11 | BG | 1000+ | C, P | ST | PA |  | PR | PR | L |
| **Heelan and Eisenmann 2006**(22) | USA | 5-11 | B & G | <100 | C | ST | MVPA |  | PR | OB | L |
| **Henderson et al. 2012**(23) | Canada | 5-11 | BG | 101-500 | C | SB, ST | MVPA |  | OB, SR | OB | H |
| **Huston et al. 1999**(24) | USA | 0-5 | BG | 101-500 | P | TV | P |  | PR | PR | L |
| **Jago et al. 2005**(25) | USA | 0-5 | BG | 101-500 | C | SB, TV | PAHR, PA |  | OB | OB | H |
| **Jago et al. 2005**(26) | USA | 0-5 | BG | 101-500 | C | SB, TV | PAHR, PA |  | OB | OB | H |
| **Jollie-Trottier et al. 2009**(27) | USA | 5-11 | BG | 101-500 | C | TV | PA |  | SR | SR | L |
| **King et al.**  **2011**(28) | UK | 5-11 | BG | 101-500 | C | SB | AT |  | PR | PR | H |
| **Kourlaba et al. 2009**(29) | Greece | 0-5 | BG | 1000+ | C | ST | PA |  | PR | PR | L |
| **Laurson et al. 2008**(30) | USA | 5-11 | B & G | 501-1000 | C | ST, TV, VG | STEPS |  | SR | OB | H |
| **Lazarou and Soteriades 2009**(31) | Cyprus | 5-11 | BG | 1000+ | C | H, ST, VG | PA, SP |  | SR | SR | L |
| **Lindquist et al. 1999**(32) | USA | 5-11 | BG | 101-500 | C | TV | EX, PE |  | PR | PR | L |
| **Manios et al. 2004**(33) | Greece | 5-11 | BG | 101-500 | C | ST | MVPA |  | SR | SR | L |
| **McCormack et al. 2011†**(34) | Australia | 5-11 | BG | 501-1000 | C | LOW ST | STEPS |  | SR | OB | H |
| **McKenzie et al. 2008**(35) | USA | 5-11 | BG | 101-500 | C | ST | EE, MVPA |  | OB | OB | H |
| **Mitchell et al. 2009**(36) | UK | 5-11 | BG | 1000+ | C | SB | MVPA |  | OB | OB | H |
| **Montgomery et al. 2004**(37) | UK | 5-11 | BG | 101-500 | C | SB | EE, PA |  | OB | OB | H |
| **Morgan et al. 2008**(38) | Australia | 5-11 | B & G | <100 | C | ST | CPM, MPA, VPA |  | PR | OB | H |
| **Oliver et al. 2011**(39) | NZ | 5-11 | BG | 101-500 | C | SB, TV, SITTING | LPA, MPA, VPA |  | OB, PR | OB | H |
| **Ortileb et al. 2013**(40) | Germany | 5-11 | BG | 1000+ | C | ST | MPA, VPA |  | PR | PR | H |
| **Pagani et al. 2010**(41) | Canada | 0-5 | BG | 1000+ | P | TV | PA |  | PR | PR | L |
| **Page et al.**  **2010**(42) | UK | 5-11 | BG | 1000+ | C | SB, C, TV | MVPA |  | OB, PR | OB | H |
| **Pate et al.**  **1997†**(43) | USA | 5-11 | BG | 101-500 | C | ST | LOW PA |  | SR | SR | H |
| **Pearce et al. 2012**(44) | UK | 5-11 | BG | 101-500 | C | SB | SP |  | OB | SR | H |
| **Peneau et al. 2011**(45) | France | 5-11 | BG | 1000+ | C | TV | AT, OG |  | PR | PR | L |
| **Perez et al. 2003**(46) | Canada | 5-11 | BG | 1000+ | P | TV, VG | PA |  | PR | PR | H |
| **Perez-Rodriguez et al. 2012**(47) | Mexico | 5-11 | BG | 101-500 | C | TV | LPA, MPA |  | PR | OB | L |
| **Racine et al. 2011**(48) | USA | 5-11 | G | 1000+ | C | ST | PA |  | SR | SR | L |
| **Roemmich et al. 2006**(49) | USA | 5-11 | BG | <100 | C | TV | CPM |  | OB | OB | H |
| **Sallis et al.**  **1993**(50) | USA | 0-5 | BG | 101-500 | C | TV | PA |  | SR | OB | H |
| **Salmon et al. 2006**(51) | Australia | 5-11 | BG | 1000+ | C | TV | MVPA, PA |  | PR | OB, PR | H |
| **Sardinha et al. 2008**(52) | Portugal | 5-11 | BG | 101-500 | C | SB | LPA, MVPA |  | OB | OB | H |
| **Simons-Morton et al. 1997**(53) | USA | 5-11 | BG | 1000+ | C | SB | MVPA, VPA |  | SR | SR | H |
| **Smith et al. 2008**(54) | Canada | 5-11 | BG | 101-500 | C | ST | PA |  | SR | SR | H |
| **Spinks et al. 2006†**(55) | Australia | 5-11 | BG | 501-1000 | C | ST | I |  | SR | PR | L |
| **Stettler et al. 2004**(56) | Switzerland | 5-11 | BG | 501-1000 | C | TV, VG | PA |  | SR | TR | L |
| **Straker et al. 2006**(57) | Australia | 0-5 | BG | 1000+ | C | C | PA |  | PR | PR | L |
| **Tanasescu et al. 2000**(58) | USA | 5-11 | B & G | 101-500 | C | TV | PA |  | PR | SR | L |
| **te Velde et al. 2007**(59) | Multiple | 5-11 | B & G | 1000+ | C | C, TV | EX |  | SR | SR | H |
| **Trost et al.**  **1996**(60) | USA | 5-11 | BG | 101-500 | C | TV | MVPA, VPA |  | SR | SR | L |
| **Trost et al.**  **1999**(61) | USA | 5-11 | B & G | 101-500 | C | ST | MPA, VPA |  | SR | OB | L |
| **Trudeau et al. 2004**(62) | Canada | 5-11 | BG | 101-500 | C | TV, SB | PA |  | SR | SR | L |
| **Vandewater et al. 2004**(63) | USA | 5-11 | BG | 1000+ | C | C, R | MPA, VPA |  | PR | PR | L |
| **Vandewater et al. 2006**(64) | USA | 0-5, 5-11 | BG | 1000+ | C | TV | AP |  | PR | PR | H |
| **Wilkin et al. 2006**(65) | UK | 0-5 | B & G | 501-1000 | C | ST | LPA, PA |  | PR | OB | H |

0-5=children aged 0-5 years, 5-11=children aged 5-11 years; B=boys only, G=girls only, BG=boys and girls combined, B&G=boys and girls analysed separately; C=cross-sectional, P=prospective; SR=self-report, PR=parent-report, OB=objective; L=low quality, H=high quality

TV=television viewing, VG=video games, C=computer, ST=screen time, SB=sedentary behaviour, R=reading, H=homework, In=internet use, PA=physical activity, SP=sport, MVPA=moderate-to-vigorous physical activity, MPA=moderate physical activity, VPA=vigorous physical activity, LPA=light physical activity, HPA=hard physical activity, LTPA=leisure-time physical activity, EX=exercise, AT=active travel, SAL=sport and active leisure, EE=energy=expenditure, P=Play, OPA=organised physical activity, CPM=counts per minute, PAHR=physical activity heart rate

**†** Included in the analysis for associations between sedentary behaviour and inactivity or low sedentary behaviour and physical activity

**References**

1. Adams A, Prince R. Correlates of physical activity in young American Indian children: lessons learned from the Wisconsin Nutrition and Growth Study. *J Public Health Manag Pract* 2010;**16**:394–400.

2. Anderson CB, Hughes SO, Fuemmeler BF. Parent-child attitude congruence on type and intensity of physical activity: testing multiple mediators of sedentary behavior in older children. *Health Psychol* 2009;**28**:428–38.

3. Anderson SE, Economos CD, Must A. Active play and screen time in US children aged 4 to 11 years in relation to sociodemographic and weight status characteristics: a nationally representative cross-sectional analysis. *BMC Public Health* 2008;**8**:366.

4. Bergh IH, Grydeland M, Bjelland M, et al. Personal and social-environmental correlates of objectively measured physical activity in Norwegian pre-adolescent children. *Scand J Med Sci Sports* 2011;**21**:e315–24.

5. Brophy S, Cooksey R, Lyons RA, Thomas NE, Rodgers SE, Gravenor MB. Parental factors associated with walking to school and participation in organised activities at age 5: analysis of the Millennium Cohort Study. *BMC Public Health* 2011;**11**:14.

6. Brown JE, Broom DH, Nicholson JM, Bittman M. Do working mothers raise couch potato kids? Maternal employment and children’s lifestyle behaviours and weight in early childhood. *Soc Sci Med* 2010;**70**:1816–24.

7. Brown JE, Nicholson JM, Broom DH, Bittman M. Television Viewing by School-Age Children: Associations with Physical Activity, Snack Food Consumption and Unhealthy Weight. *Soc Indic Res* 2010;**101**:221–5.

8. Byun W, Dowda M, Pate RR. Correlates of objectively measured sedentary behavior in US preschool children. *Pediatrics* 2011;**128**:937–45.

9. Calderon LL, Johnston PK, Lee JW, Haddad EH. Risk factors for obesity in Mexican-American girls: dietary factors, anthropometric factors, and physical activity. *J Am Diet Assoc* 1996;**96**:1177–9.

10. Carandente F, Roveda E, Montaruli A, Pizzini G. Nutrition, activity behavior and body constitution in primary school children. *Biol Sport* 2009;**26**:349–67.

11. Cox R, Skouteris H, Rutherford L, Fuller-Tyszkiewicz M, Dell’ Aquila D, Hardy LL. Television viewing, television content, food intake, physical activity and body mass index: a cross-sectional study of preschool children aged 2-6 years. *Health Promot J Austr* 2012;**23**:58–62.

12. Drenowatz C, Carlson JJ, Pfeiffer KA, Eisenmann JC. Joint association of physical activity/screen time and diet on CVD risk factors in 10-year-old children. *Front Med* 2012;**6**:428–35.

13. Dumith SC, Hallal PC, Menezes AMB, Araújo CL. Sedentary behavior in adolescents: the 11-year follow-up of the 1993 Pelotas (Brazil) birth cohort study. *Cad Saude Publica* 2010;**26**:1928–36.

14. Dumith SC, Gigante DP, Domingues MR, Hallal PC, Menezes AMB, Kohl HW. Predictors of physical activity change during adolescence: a 3.5-year follow-up. *Public Health Nutr* 2012;**15**:2237–45.

15. DuRant RH, Baranowski T, Johnson M, Thompson WO. The relationship among television watching, physical activity, and body composition of young children. *Pediatrics* 1994;**94**:449–55.

16. Ekstedt M, Nyberg G, Ingre M, Ekblom Ö, Marcus C. Sleep, physical activity and BMI in six to ten-year-old children measured by accelerometry: a cross-sectional study. *Int J Behav Nutr Phys Act* 2013;**10**:82.

17. Esmaeilzadeh S, Kalantari H-A, Nakhostin-Roohi B. Cardiorespiratory fitness, activity level, health-related anthropometric variables, sedentary behaviour and socioeconomic status in a sample of Iranian 7-11 year old boys. *Biol Sport* 2013;**30**:67–71.

18. Foley JT, McCubbin JA. An exploratory study of after-school sedentary behaviour in elementary school-age children with intellectual disability. *J Intellect Dev Disabil* 2009;**34**:3–9.

19. Fulton JE, Dai S, Steffen LM, Grunbaum JA, Shah SM, Labarthe DR. Physical activity, energy intake, sedentary behavior, and adiposity in youth. *Am J Prev Med* 2009;**37**:S40–9.

20. Hager RL. Television viewing and physical activity in children. *J Adolesc Health* 2006;**39**:656–61.

21. Hands BP, Chivers PT, Parker HE, Beilin L, Kendall G, Larkin D. The associations between physical activity, screen time and weight from 6 to 14 yrs: the Raine Study. *J Sci Med Sport* 2011;**14**:397–403.

22. Heelan KA, Eisenmann JC. Physical Activity, Media Time, and Body Composition in Young Children. *J Phys Act Health* 2006;**3**:200–9.

23. Henderson M, Gray-Donald K, Mathieu M-E, et al. How are physical activity, fitness, and sedentary behavior associated with insulin sensitivity in children? *Diabetes Care* 2012;**35**:1272–8.

24. Huston AC, Wright JC, Marquis J, Green SB. How young children spend their time: Television and other activities. *Dev Psychol* 1999;**35**:912–25.

25. Jago R, Baranowski T, Baranowski JC, Thompson D, Greaves KA. BMI from 3-6 y of age is predicted by TV viewing and physical activity, not diet. *Int J Obes* 2005;**29**:557–64.

26. Jago R, Baranowski T, Thompson D, Baranowski J, Greaves KA. Sedentary behavior, not TV viewing, predicts physical activity among 3-to 7-year-old children. *Pediatr Exerc Sci* 2005;**17**:364–76.

27. Jollie-Trottier T, Holm JE, McDonald JD. Correlates of overweight and obesity in american Indian children. *J Pediatr Psychol* 2009;**34**:245–53.

28. King AC, Parkinson KN, Adamson AJ, et al. Correlates of objectively measured physical activity and sedentary behaviour in English children. *Eur J Public Health* 2011;**21**:424–31.

29. Kourlaba G, Kondaki K, Liarigkovinos T, Manios Y. Factors associated with television viewing time in toddlers and preschoolers in Greece: the GENESIS study. *J Public Health* 2009;**31**:222–30.

30. Laurson KR, Eisenmann JC, Welk GJ, Wickel EE, Gentile DA, Walsh DA. Combined influence of physical activity and screen time recommendations on childhood overweight. *J Pediatr* 2008;**153**:209–14.

31. Lazarou C, Soteriades ES. Physical activity patterns among preadolescent children in Cyprus: The CYKIDS study. *J Phys Act Health* 2009;**6**:185–94.

32. Lindquist CH, Reynolds KD, Goran MI. Sociocultural determinants of physical activity among children. *Prev Med* 1999;**29**:305–12.

33. Manios Y, Yiannakouris N, Papoutsakis C, et al. Behavioral and physiological indices related to BMI in a cohort of primary schoolchildren in Greece. *Am J Hum Biol* 2004;**16**:639–47.

34. McCormack GR, Giles-Corti B, Timperio A, Wood G, Villanueva K. A cross-sectional study of the individual, social, and built environmental correlates of pedometer-based physical activity among elementary school children. *Int J Behav Nutr Phys Act* 2011;**8**:30.

35. McKenzie TL, Baquero B, Crespo NC, Arredondo EM, Campbell NR, Elder JP. Environmental correlates of physical activity in Mexican American children at home. *J Phys Act Health* 2008;**5**:579–91.

36. Mitchell JA, Mattocks C, Ness AR, et al. Sedentary behavior and obesity in a large cohort of children. *Obesity* 2009;**17**:1596–602.

37. Montgomery C, Reilly JJ, Jackson DM, et al. Relation between physical activity and energy expenditure in a representative sample of young children. *Am J Clin Nutr* 2004;**80**:591–6.

38. Morgan PJ, Okely AD, Cliff DP, Jones RA, Baur LA. Correlates of objectively measured physical activity in obese children. *Obesity* 2008;**16**:2634–41.

39. Oliver M, Schluter PJ, Rush E, Schofield GM, Paterson J. Physical activity, sedentariness, and body fatness in a sample of 6-year-old Pacific children. *Int J Pediatr Obes* 2011;**6**:e565–73.

40. Ortlieb S, Schneider G, Koletzko S, et al. Physical activity and its correlates in children: a cross-sectional study (the GINIplus & LISAplus studies). *BMC Public Health* 2013;**13**:349.

41. Pagani LS, Fitzpatrick C, Barnett TA, Dubow E. Prospective associations between early childhood television exposure and academic, psychosocial, and physical well-being by middle childhood. *Arch Pediatr Adolesc Med* 2010;**164**:425–31.

42. Page AS, Cooper AR, Griew P, Jago R. Children’s screen viewing is related to psychological difficulties irrespective of physical activity. *Pediatrics* 2010;**126**:e1011–7.

43. Pate RR, Trost SG, Felton GM, Ward DS, Dowda M, Saunders R. Correlates of physical activity behavior in rural youth. *Res Q Exerc Sport* 1997;**68**:241–8.

44. Pearce MS, Basterfield L, Mann KD, et al. Early predictors of objectively measured physical activity and sedentary behaviour in 8-10 year old children: the Gateshead Millennium Study. *PLoS One* 2012;**7**:e37975.

45. Péneau S, Salanave B, Rolland-Cachera M-F, Hercberg S, Castetbon K. Correlates of sedentary behavior in 7 to 9-year-old French children are dependent on maternal weight status. *Int J Obes* 2011;**35**:907–15.

46. Pérez CE. Children who become active. *Health Reports* 2003;**14**:17–28.

47. Perez-Rodriguez M, Melendez G, Nieto C, Aranda M, Pfeffer F. Dietary and physical activity/inactivity factors associated with obesity in school-aged children. *Adv Nutr* 2012;**3**:622S–628S.

48. Racine EF, DeBate RD, Gabriel KP, High RR. The relationship between media use and psychological and physical assets among third- to fifth-grade girls. *J Sch Health* 2011;**81**:749–55.

49. Roemmich JN, Epstein LH, Raja S, Yin L, Robinson J, Winiewicz D. Association of access to parks and recreational facilities with the physical activity of young children. *Prev Med* 2006;**43**:437–41.

50. Sallis JF, Nader PR, Broyles SL, et al. Correlates of physical activity at home in Mexican-American and Anglo-American preschool children. *Health Psychol* 1993;**12**:390–8.

51. Salmon J, Campbell KJ, Crawford DA. Television viewing habits associated with obesity risk factors: a survey of Melbourne schoolchildren. *Med J Aust* 2006;**184**:64–7.

52. Sardinha LB, Andersen LB, Anderssen SA, et al. Objectively measured time spent sedentary is associated with insulin resistance independent of overall and central body fat in 9- to 10-year-old Portuguese children. *Diabetes Care* 2008;**31**:569–75.

53. Simons-Morton BG, McKenzie TJ, et al. Physical activity in a multiethnic population of third graders in four states. *Am J Public Health* 1997;**87**:45–50.

54. Smith NEI, Rhodes RE, Naylor P-J, McKay HA. Exploring moderators of the relationship between physical activity behaviors and television viewing in elementary school children. *Am J Health Promot* 2008;**22**:231–6.

55. Spinks A, Macpherson A, Bain C, McClure R. Determinants of sufficient daily activity in Australian primary school children. *J Paediatr Child Health* 2006;**42**:674–9.

56. Stettler N, Signer TM, Suter PM. Electronic games and environmental factors associated with childhood obesity in Switzerland. *Obes Res* 2004;**12**:896–903.

57. Straker LM, Pollock CM, Zubrick SR, Kurinczuk JJ. The association between information and communication technology exposure and physical activity, musculoskeletal and visual symptoms and socio-economic status in 5-year-olds. *Child Care Health Dev* 2006;**32**:343–51.

58. Tanasescu M, Ferris AM, Himmelgreen DA, Rodriguez N, Pérez-Escamilla R. Biobehavioral factors are associated with obesity in Puerto Rican children. *J Nutr* 2000;**130**:1734–42.

59. Te Velde SJ, De Bourdeaudhuij I, Thorsdottir I, et al. Patterns in sedentary and exercise behaviors and associations with overweight in 9-14-year-old boys and girls--a cross-sectional study. *BMC Public Health* 2007;**7**:16.

60. Trost SG, Pate RR, Dowda M, Saunders R, Ward DS, Felton G. Gender differences in physical activity and determinants of physical activity in rural fifth grade children. *J Sch Health* 1996;**66**:145–50.

61. Trost SG, Pate RR, Ward DS, Saunders R, Riner W. Correlates of objectively measured physical activity in preadolescent youth. *Am J Prev Med* 1999;**17**:120–6.

62. Trudeau FF, Laurencelle L, Shephard RJ. Tracking of physical activity from childhood to adulthood. *Med Sci Sports Exerc* 2004;**36**:1937–43.

63. Vandewater EA, Shim M, Caplovitz AG. Linking obesity and activity level with children’s television and video game use. *J Adolesc* 2004;**27**:71–85.

64. Vandewater EA, Bickham DS, Lee JH. Time well spent? Relating television use to children’s free-time activities. *Pediatrics* 2006;**117**:e181–91.

65. Wilkin TJ, Mallam KM, Metcalf BS, Jeffery AN, Voss LD. Variation in physical activity lies with the child, not his environment: evidence for an “activitystat” in young children (EarlyBird 16). *Int J Obes* 2006;**30**:1050–5.
