# Supplementary material for: Associations between sedentary behaviour and physical activity in children and adolescents: a meta-analysis
Source: Obes Rev. 2014 May 20;15(8):666–75. doi: 10.1111/obr.12188 (PMC4282352; doi:10.1111/obr.12188)
Supplement: Supplementary file 2 — Table S2. Descriptive characteristics of studies including children and adolescents. [file obr0015-0666-sd2.docx]

**Supplementary Table 2.** Descriptive characteristics of studies including children and adolescents.

| **Author** | **Country** | **Age group** | **Gender** | **Sample size** | **Study design** | **Exposures / Outcomes** | |  | **Assessment method** | | **Quality rating** |
| --- | --- | --- | --- | --- | --- | --- | --- | --- | --- | --- | --- |
|  |  |  |  |  |  | **SB** | **PA** |  | **SB** | **PA** |  |
| **Carlson et al. 2010***(1) | USA | C&A | BG | 1000+ | C | ST | PA, OPA |  | SR | SR | L |
| **Cummings et al. 2007**(2) | USA | C&A | B & G | 1000+ | C | VG | SAL |  | SR | SR | L |
| **De Jong et al. 2013**(3) | Netherlands | C&A | B & G | 1000+ | C | C, TV | SP, P |  | SR | SR | L |
| **Ekelund et al. 2012**(4) | Multiple | C&A | BG | 1000+ | C | SB | CPM |  | OB | OB | H |
| **Ekelund et al. 2006**(5) | Multiple | C&A | BG | 1000+ | C | TV | PA |  | OB | SR | H |
| **Fernandes et al. 2008**(6) | Brazil | C&A | BG | 1000+ | C | TV | SP, C, W |  | SR | SR | H |
| **Katzmarzyk et al. 1998**(7) | USA | C&A | B & G | 101-500 | C | TV | EE |  | SR | SR | L |
| **Myers et al. 1996**(8) | USA | C&A | BG | 1000+ | C | SB | PA |  | SR | SR | L |
| **Ogunleye et al. 2011†**(9) | UK | C&A | BG | 1000+ | C | LOW ST | PA |  | SR | SR | H |
| **Olds et al.**  **2006**(10) | Australia | C&A | BG | 1000+ | C | ST, VG | MVPA, PA |  | SR | SR | H |
| **Olds et al.**  **2011**(11) | Australia | C&A | BG | 1000+ | C | ST | MVPA |  | SR | SR | H |
| **Singh et al. 2008**(12) | USA | C&A | BG | 1000+ | C | C, TV | VPA |  | SR | SR | L |
| **Tudor-Locke et al. 2011**(13) | USA | C&A | B & G | 1000+ | C | TV | STEPS |  | SR | OB | H |

C&A=children and adolescents; B=boys only, G=girls only, BG=boys and girls combined, B&G=boys and girls analysed separately; C=cross-sectional, P=prospective; SR=self-report, PR=parent-report, OB=objective; L=low quality, H=high quality

TV=television viewing, VG=video games, C=computer, ST=screen time, SB=sedentary behaviour, R=reading, H=homework, In=internet use, PA=physical activity, SP=sport, MVPA=moderate-to-vigorous physical activity, MPA=moderate physical activity, VPA=vigorous physical activity, LPA=light physical activity, HPA=hard physical activity, LTPA=leisure-time physical activity, EX=exercise, AT=active travel, SAL=sport and active leisure, EE=energy expenditure, P=Play, OPA=organised physical activity, CPM=counts per minute, PAHR=physical activity heart rate

* Included in the analysis for the association between physical activity (exposure) and sedentary behaviour (outcome); **†** Included in the analysis for associations between sedentary behaviour and inactivity or low sedentary behaviour and physical activity

**References**

1. Carlson SA, Fulton JE, Lee SM, Foley JT, Heitzler C, Huhman M. Influence of limit-setting and participation in physical activity on youth screen time. *Pediatrics* 2010;**126**:e89–96.

2. Cummings HM, Vandewater EA. Relation of adolescent video game play to time spent in other activities. *Arch Pediatr Adolesc Med* 2007;**161**:684–9.

3. De Jong E, Visscher TLS, HiraSing R a, Heymans MW, Seidell JC, Renders CM. Association between TV viewing, computer use and overweight, determinants and competing activities of screen time in 4- to 13-year-old children. *Int J Obes* 2011;**37**:47–53.

4. Ekelund U, Luan J, Sherar LB, Esliger DW, Griew P, Cooper A. Moderate to vigorous physical activity and sedentary time and cardiometabolic risk factors in children and adolescents. *JAMA* 2012;**307**:704–12.

5. Ekelund U, Brage S, Froberg K, et al. TV viewing and physical activity are independently associated with metabolic risk in children: the European Youth Heart Study. *PLoS Med* 2006;**3**:e488.

6. Fernandes RA, Júnior IFF, Cardoso JR, Vaz Ronque ER, Loch MR, de Oliveira AR. Association between regular participation in sports and leisure time behaviors in Brazilian adolescents: a cross-sectional study. *BMC Public Health* 2008;**8**:329.

7. Katzmarzyk PT, Malina RM, Song TM, Bouchard C. Television viewing, physical activity, and health-related fitness of youth in the Québec Family Study. *J Adolesc Health* 1998;**23**:318–25.

8. Myers L, Strikmiller PK, Webber LS, Berenson GS. Physical and sedentary activity in school children grades 5-8: the Bogalusa Heart Study. *Med Sci Sports Exerc* 1996;**28**:852–9.

9. Ogunleye a a, Voss C, Sandercock GR. Prevalence of high screen time in English youth: association with deprivation and physical activity. *J Public Health* 2011;**34**:46–53.

10. Olds T, Ridley K, Dollman J. Screenieboppers and extreme screenies: the place of screen time in the time budgets of 10-13 year-old Australian children. *Aust N Z J Public Health* 2006;**30**:137–42.

11. Olds T, Maher CA, Ridley K. The place of physical activity in the time budgets of 10- to 13-year-old Australian children. *J Phys Act Health* 2011;**8**:548–57.

12. Singh GK, Kogan MD, Siahpush M, van Dyck PC. Independent and joint effects of socioeconomic, behavioral, and neighborhood characteristics on physical inactivity and activity levels among US children and adolescents. *J Community Health* 2008;**33**:206–16.

13. Tudor-Locke C, Craig CL, Cameron C, Griffiths JM. Canadian children’s and youth's pedometer-determined steps/day, parent-reported TV watching time, and overweight/obesity: the CANPLAY Surveillance Study. *Int J Behav Nutr Phys Act* 2011;**8**:66.
