# Supplementary material for: Associations between sedentary behaviour and physical activity in children and adolescents: a meta-analysis
Source: Obes Rev. 2014 May 20;15(8):666–75. doi: 10.1111/obr.12188 (PMC4282352; doi:10.1111/obr.12188)
Supplement: Supplementary file 3 — Table S3. Descriptive characteristics of studies including adolescents aged 12–18 years. [file obr0015-0666-sd3.docx]

**Supplementary Table 3.** Descriptive characteristics of studies including adolescents aged 12-18 years

| **Author** | **Country** | **Age group** | **Gender** | **Sample size** | **Study design** | **Exposures / Outcomes** | |  | **Assessment method** | | **Quality rating** |
| --- | --- | --- | --- | --- | --- | --- | --- | --- | --- | --- | --- |
|  |  |  |  |  |  | **SB** | **PA** |  | **SB** | **PA** |  |
| **Al-Hazzaa et al. 2011**(1) | Saudi Arabia | OA | B & G | 1000+ | C | ST | PA, VPA |  | SR | SR | L |
| **Al-Nakeeb et al. 2012**(2) | Multiple | OA | BG | 1000+ | C | C, SB, TV | PA |  | SR | SR | L |
| **Babey et al. 2012***(3) | USA | YA | BG | 1000+ | C | C, TV | PA |  | SR | SR | L |
| **Barbosa Filho et al. 2012***(4) | Brazil | YA | BG | 1000+ | C | TV | PA, PE |  | SR | SR | L |
| **Biddle et al. 2009**(5) | UK | YA | B & G | 1000+ | C | TV | AT, SE |  | SR | SR | L |
| **Bratteby et al. 2005**(6) | Sweden | YA | BG | 101-500 | C | SB | PA |  | SR | SR | H |
| **Brodersen et al. 2005**(7) | USA | YA | B & G | 1000+ | C | ST | PA |  | SR | SR | L |
| **Bungum and Vincent 1997**(8) | USA | A | G | 501-1000 | C | TV | PA |  | SR | SR | L |
| **Burke et al. 2006**(9) | Australia | YA | B & G | 101-500 | C | C, TV | MPA |  | SR | SR | L |
| **Ceschini et al. 2009†**(10) | Brazil | YA | BG | 1000+ | C | TV | I |  | SR | SR | L |
| **Chen et al.**  **2007**(11) | Taiwan | A | BG | 1000+ | C | SB | PA |  | SR | SR | L |
| **Chen et al.**  **2008**(12) | Taiwan | YA | BG | 501-1000 | C | C, TV | EX |  | SR | SR | L |
| **Cuenca-Garcia et al. 2013**(13) | Multiple | YA | BG | 1000+ | C | H, ST | MVPA |  | SR | SR | L |
| **Dalton et al. 2011**(14) | USA | YA | BG | 101-500 | C | ST | PA |  | SR | SR | L |
| **Denton et al. 2013**(15) | UK | YA | B & G | 101-500 | C | SB | HPA, VPA, MPA, LPA |  | OB | OB | H |
| **Devís-Devís et al. 2010**(16) | Spain | YA | BG | 101-500 | C | C, TV | LPA, MPA |  | SR | SR | L |
| **Dumith et al. 2010**(17) | Brazil | YA | B & G | 1000+ | C | C, TV, VG | PA |  | SR | SR | L |
| **Elgar et al.**  **2005**(18) | UK | YA | BG | 101-500 | P | ST | EX |  | SR | SR | L |
| **Farias Junior et al. 2011†**(19) | Brazil | OA | B & G | 1000+ | C | LOW C, LOW TV | PA |  | SR | SR | L |
| **Feldman et al. 2003**(20) | Canada | YA | B & G | 1000+ | C | C, TV, VG | PA |  | SR | SR | L |
| **Fermino et al. 2010**(21) | Brazil | OA | BG | 1000+ | C | C, TV | MVPA |  | SR | SR | L |
| **Gebremariam et al. 2013**(22) | Norway | YA | BG | 501-1000 | P | C, TV | LTPA |  | SR | SR | H |
| **Gorely et al. 2007**(23) | UK | YA | B & G | 1000+ | C | C, TV | SP |  | SR | SR | L |
| **Hanson and Chen 2007**(24) | USA | OA | BG | 101-500 | C | SB | PA |  | SR | SR | L |
| **Hearst et al. 2012**(25) | USA | YA | B & G | 101-500 | P | ST | MVPA |  | SR | OB | H |
| **Ho and Lee 2001**(26) | China | YA | B & G | 1000+ | C | C, IN, VG | EX |  | SR | SR | L |
| **Hohepa et al. 2009**(27) | NZ | YA | BG | 1000+ | C | TV | PA |  | SR | SR | L |
| **Hong et al.**  **2009**(28) | China | YA | BG | 1000+ | C | TV | PA |  | SR | SR | L |
| **Hsu et al.**  **2011**(29) | USA | YA | BG | 101-500 | C | SB | MVPA |  | SR | OB | H |
| **Iannotti et al. 2009**(30) | Multiple | YA | BG | 1000+ | C | ST | PA |  | SR | SR | H |
| **Jaakkola et al. 2009**(31) | Finland | YA | B & G | 101-500 | C | ST | LTPA |  | SR | SR | L |
| **Jago et al.**  **2006**(32) | USA | YA | B | 101-500 | C | SB | LPA |  | OB | OB | H |
| **Jago et al.**  **2007**(33) | USA | YA | B | 101-500 | C | SB | LPA, MVPA |  | OB, SR | OB | H |
| **Janz and Mahoney 1997**(34) | USA | YA | B & G | 101-500 | C | VG | CPM, MPA, VPA |  | SR | OB | H |
| **Kaluski et al. 2009†**(35) | Israel | A | B & G | 1000+ | C | LOW SB | MVPA |  | SR | SR | L |
| **Karaca et al. 2011**(36) | Turkey | YA | BG | 501-1000 | C | ST | SP |  | SR | SR | L |
| **Katzmarzyk and Malina 1998**(37) | USA | YA | B & G | 101-500 | C | TV | SP |  | SR | SR | L |
| **Kerner et al. 2004**(38) | USA | YA | G | 101-500 | C | IN, SB, TV | LTPA |  | SR | SR | L |
| **Koezuka et al. 2006†**(39) | Canada | OA | B & G | 1000+ | C | R, C, VG, TV | I |  | SR | SR | L |
| **Lajunen et al. 2009**(40) | USA | A | B & G | 1000+ | P | SB | PA |  | SR | SR | H |
| **Landsberg et al. 2010**(41) | Germany | YA | B & G | 1000+ | C | ST | LTPA |  | SR | SR | L |
| **Lasserre et al. 2007**(42) | Switzerland | YA | BG | 1000+ | C | TV, VG | LTPA |  | SR | SR | L |
| **Leatherdale et al. 2008**(43)**^#^** | USA | A | BG | 1000+ | C | H, R, ST | MPA, LOW PA |  | SR | SR | L |
| **Leatherdale et al. 2010**(44) | Canada | YA | BG | 1000+ | C | HPA, MPA | ST |  | SR | SR | L |
| **Lippo et al. 2010†**(45) | Brazil | OA | BG | 501-1000 | C | C, TV | I |  | SR | SR | L |
| **Lobelo et al. 2009**(46) | USA | YA | G | 1000+ | C | ST | MVPA, VPA |  | SR | SR | L |
| **Lowry et al. 2002†**(47) | USA | OA | B & G | 1000+ | C | TV | I |  | SR | SR | L |
| **Maher et al. 2012**(48) | Australia | YA | B & G | 1000+ | C | ST | MVPA |  | SR | SR | L |
| **Mamum et al. 2012**(49) | Australia | YA | BG | 1000+ | C | TV | SP |  | SR | SR | L |
| **Mandic et al. 2012**(50) | NZ | YA | BG | 1000+ | C | ST | SP |  | SR | SR | L |
| **Marshall et al. 2002**(51) | Multiple | YA | BG | 1000+ | C | C, TV, VG | PA |  | SR | SR | L |
| **Martinez-Gomez et al. 2011**(52) | Spain | YA | BG | 101-500 | C | SB | MVPA |  | OB | OB | H |
| **Martinez-Gomez et al. 2011**(53) | Spain | A | BG | 1000+ | C | TV | AT |  | SR | SR | L |
| **McGuire et al. 2002**(54) | USA | A | BG | 1000+ | C | TV | LTPA |  | SR | SR | H |
| **Melkevik et al. 2010**(55) | Multiple | YA | B & G | 1000+ | C | C, ST, TV, VG | MVPA, LPA |  | SR | SR | H |
| **Mota et al.**  **2008**(56) | Portugal | YA | B & G | 1000+ | C | ST | LTPA |  | SR | SR | H |
| **Motl et al.**  **2006**(57) | USA | YA | BG | 1000+ | P | TV,VG | PA |  | SR | SR | L |
| **Norman et al. 2005**(58) | USA | YA | BG | 501-1000 | C | SB | VPA |  | SR | OB | H |
| **Pate et al.**  **1996**(59) | USA | A | BG | 1000+ | C | ST | PA |  | SR | SR | L |
| **Raudsepp et al. 2008**(60) | Estonia | YA | BG | 101-500 | P | ST, SB | PA |  | SR | SR | L |
| **Rivera et al. 2010**(61) | Brazil | YA | BG | 1000+ | C | TV | PA |  | SR | SR | L |
| **Robinson et al. 1993**(62) | USA | YA | G | 501-1000 | C, P | TV | PA |  | SR | SR | L |
| **Robinson and Killen 1995**(63) | USA | A | B & G | 1000+ | C | TV | PA |  | SR | SR | L |
| **Ruiz et al.**  **2010**(64) | Spain | A | BG | 1000+ | C | TV, VG | PA |  | SR | SR | L |
| **Sandercock et al. 2012**(65) | UK | YA | B & G | 1000+ | C | ST | PA, PE, SP |  | SR | SR | H |
| **Schneider et al. 2007**(66) | USA | YA | G | 101-500 | C | SB | MPA, VPA |  | SR | SR | L |
| **Serrano-Sanchez et al 2011**(67) | Spain | A | BG | 1000+ | C | ST | PA |  | SR | SR | H |
| **Sirard et al. 2008**(68) | USA | YA | G | 1000+ | C | SB, TV | MVPA, VPA |  | SR | SR | L |
| **Sjolie and Thuen 2002**(69) | Norway | YA | BG | <100 | C | R, ST | LTPA |  | SR | SR | L |
| **Snoek et al. 2006**(70) | Netherlands | YA | B & G | 1000+ | C | ST | PA |  | SR | SR | L |
| **Spink et al. 2005†**(71) | Canada | A | BG | 1000+ | C | LOW SB | PA |  | SR | SR | H |
| **Strauss et al. 2001**(72) | USA | YA | BG | <100 | C | SB | MPA, VPA |  | SR | OB | L |
| **Strong et al. 2012**(73) | USA | YA | B & G | 1000+ | C | TV | MVPA, VPA, SP |  | SR | SR | H |
| **Tammelin et al. 2007**(74) | Finland | OA | B & G | 1000+ | C | C, R, SB, TV | PA |  | SR | SR | L |
| **Taveras et al. 2007**(75) | USA | YA | B & G | 1000+ | P | SB, TV | MVPA |  | SR | SR | L |
| **Taverno Ross et al. 2013**(76) | USA | YA | G | 1000+ | C | SB | PA |  | SR | SR | L |
| **Trang et al. 2009†**(77) | Vietnam | YA | B & G | 1000+ | C | TV, VG | I |  | SR | SR | H |
| **Ussher et al. 2007**(78) | UK | YA | BG | 1000+ | C | SB | PA |  | SR | SR | L |
| **Van den Bulck et al. 2009**(79) | Belgium | YA | BG | 1000+ | C, P | TV | EX |  | SR | SR | L |
| **van der Sluis et al. 2010†**(80) | Norway | YA | B & G | 1000+ | P | ST | LOW PA |  | SR | SR | L |
| **Vilhjalmsson et al. 1998**(81) | Iceland | A | BG | 1000+ | C | C, TV | PA |  | SR | SR | L |
| **Wang et al. 2006**(82) | USA | YA | BG | 501-1000 | C | C, TV | PA |  | SR | SR | L |
| **Webb et al. 2013**(83) | UK | YA | G | 101-500 | C | SB | MVPA |  | SR | SR | L |
| **Williams and Mummery 2011**(84) | Australia | YA | B & G | 1000+ | C | H, ST | MVPA |  | SR | SR | L |
| **Wolf et al.**  **1993**(85) | USA | YA | BG | 501-1000 | C | TV | PA |  | SR | SR | L |
| **Zabinski et al.2007**(86) | USA | YA | BG | 501-1000 | C | SB | MVPA, VPA |  | SR | OB | L |

A=adolescents (12-18 years), YA=younger adolescents (12-15 years), OA=older adolescents (16-18 years); B=boys only, G=girls only, BG=boys and girls combined, B&G=boys and girls analysed separately; C=cross-sectional, P=prospective; SR=self-report, PR=parent-report, OB=objective; L=low quality, H=high quality

TV=television viewing, VG=video games, C=computer, ST=screen time, SB=sedentary behaviour, R=reading, H=homework, In=internet use, PA=physical activity, SP=sport, MVPA=moderate-to-vigorous physical activity, MPA=moderate physical activity, VPA=vigorous physical activity, LPA=light physical activity, HPA=hard / high physical activity, LTPA=leisure-time physical activity, EX=exercise, AT=active travel, SAL=sport and active leisure, EE=energy=expenditure, P=Play, OPA=organised physical activity, CPM=counts per minute, PAHR=physical activity heart rate

* Included in the analysis for the association between physical activity (exposure) and sedentary behaviour (outcome); **†** Included in the analysis for associations between sedentary behaviour and inactivity or low sedentary behaviour and physical activity; **^#^** Due to the method of analysis applied, this study was not included in the meta-analysis. Results summarised in the text.

**References**

1. Al-Hazzaa HM, Abahussain NA, Al-Sobayel HI, Qahwaji DM, Musaiger AO. Physical activity, sedentary behaviors and dietary habits among Saudi adolescents relative to age, gender and region. *Int J Behav Nutr Phys Act* 2011;**8**:140.

2. Al-Nakeeb Y, Lyons M, Collins P, et al. Obesity, physical activity and sedentary behavior amongst British and Saudi youth: a cross-cultural study. *Int J Environ Res Public Health* 2012;**9**:1490–506.

3. Babey SH, Hastert T a, Wolstein J. Adolescent sedentary behaviors: correlates differ for television viewing and computer use. *J Adolesc Health* 2013;**52**:70–6.

4. Barbosa Filho VC, de Campos W, Bozza R, Lopes A da S. The prevalence and correlates of behavioral risk factors for cardiovascular health among Southern Brazil adolescents: a cross-sectional study. *BMC Pediatr* 2012;**12**:130.

5. Biddle SJH, Gorely T, Marshall SJ. Is television viewing a suitable marker of sedentary behavior in young people? *Ann Behav Med* 2009;**38**:147–53.

6. Bratteby L-E, Sandhagen B, Samuelson G. Physical activity, energy expenditure and their correlates in two cohorts of Swedish subjects between adolescence and early adulthood. *Eur J Clin Nutr* 2005;**59**:1324–34.

7. Brodersen NH, Steptoe A, Williamson S, Wardle J. Sociodemographic, developmental, environmental, and psychological correlates of physical activity and sedentary behavior at age 11 to 12. *Ann Behav Med* 2005;**29**:2–11.

8. Bungum TJ, Vincent ML. Determinants of physical activity among female adolescents. *Am J Prev Med* 1997;**13**:115–22.

9. Burke V, Beilin LJ, Durkin K, Stritzke WGK, Houghton S, Cameron CA. Television, computer use, physical activity, diet and fatness in Australian adolescents. *Int J Pediatr Obes* 2006;**1**:248–55.

10. Ceschini FL, Andrade DR, Oliveira LC, Araújo Júnior JF, Matsudo VKR. Prevalence of physical inactivity and associated factors among high school students from state’s public schools. *J Pediatr* 2009;**85**:301–6.

11. Chen L-J, Haase AM, Fox KR. Physical activity among adolescents in Taiwan. *Asia Pac J Clin Nutr* 2007;**16**:354–61.

12. Chen M-Y, Liou Y-M, Wu J-Y. The relationship between TV/computer time and adolescents’ health-promoting behavior: a secondary data analysis. *J Nurs Res* 2008;**16**:75–85.

13. Cuenca-García M, Huybrechts I, Ruiz JR, et al. Clustering of Multiple Lifestyle Behaviors and Health-related Fitness in European Adolescents. *J Nutr Educ Behav* 2013;**45**:549–57.

14. Dalton WT, Schetzina KE, Pfortmiller DT, Slawson DL, Frye WS. Health behaviors and health-related quality of life among middle school children in Southern Appalachia: data from the winning with wellness project. *J Pediatr Psychol* 2011;**36**:677–86.

15. Denton SJ, Trenell MI, Plötz T, Savory LA, Bailey DP, Kerr CJ. cardiorespiratory fitness is associated with hard and light intensity physical activity but not time spent sedentary in 10-14 year old schoolchildren: the HAPPY study. *PLoS One* 2013;**8**:e61073.

16. Devís-Devís J, Peiró-Velert C, Beltrán-Carrillo VJ, Tomás JM. Brief report: Association between socio-demographic factors, screen media usage and physical activity by type of day in Spanish adolescents. *J Adolesc* 2012;**35**:213–8.

17. Dumith SC, Domingues MR, Gigante DP, Hallal PC, Menezes AMB, Kohl HW. Prevalence and correlates of physical activity among adolescents from Southern Brazil. *Rev Saude Publica* 2010;**44**:457–67.

18. Elgar FJ, Roberts C, Moore L, Tudor-Smith C. Sedentary behaviour, physical activity and weight problems in adolescents in Wales. *Public Health* 2005;**119**:518–24.

19. Farias Júnior JC de, Lopes A da S, Mota J, Hallal PC. Physical activity practice and associated factors in adolescents in Northeastern Brazil. *Rev Saude Publica* 2012;**46**:505–15.

20. Feldman DE, Barnett T, Shrier I, Rossignol M, Abenhaim L. Is physical activity differentially associated with different types of sedentary pursuits? *Arch Pediatr Adolesc Med* 2003;**157**:797–802.

21. Fermino RC, Rech CR, Hino AAF, Rodriguez Añez CR, Reis RS. Physical activity and associated factors in high-school adolescents in Southern Brazil. *Rev Saude Publica* 2010;**44**:986–95.

22. Gebremariam MK, Bergh IH, Andersen LF, et al. Are screen-based sedentary behaviors longitudinally associated with dietary behaviors and leisure-time physical activity in the transition into adolescence? *Int J Behav Nutr Phys Act* 2013;**10**:9.

23. Gorely T, Marshall SJ, Biddle SJH, Cameron N. Patterns of sedentary behaviour and physical activity among adolescents in the United Kingdom: Project STIL. *J Behav Med* 2007;**30**:521–31.

24. Hanson MD, Chen E. Socioeconomic status, race, and body mass index: the mediating role of physical activity and sedentary behaviors during adolescence. *J Pediatr Psychol* 2007;**32**:250–9.

25. Hearst MO, Patnode CD, Sirard JR, Farbakhsh K, Lytle LA. Multilevel predictors of adolescent physical activity: a longitudinal analysis. *Int J Behav Nutr Phys Act* 2012;**9**:8.

26. Ho SM., Lee TM. Computer usage and its relationship with adolescent lifestyle in Hong Kong. *J Adolesc Health* 2001;**29**:258–66.

27. Hohepa M, Scragg R, Schofield G, Kolt GS, Schaaf D. Associations Between After-School Physical Activity, Television Use, and Parental Strategies in a Sample of New Zealand Adolescents. *J Phys Act Health* 2009;**6**:299–305.

28. Hong X, Li J, Xu F, et al. Physical activity inversely associated with the presence of depression among urban adolescents in regional China. *BMC Public Health* 2009;**9**:148.

29. Hsu Y-W, Belcher BR, Ventura EE, et al. Physical activity, sedentary behavior, and the metabolic syndrome in minority youth. *Med Sci Sports Exerc* 2011;**43**:2307–13.

30. Iannotti RJ, Janssen I, Haug E, Kololo H, Annaheim B, Borraccino A. Interrelationships of adolescent physical activity, screen-based sedentary behaviour, and social and psychological health. *Int J Public Health* 2009;**54**:191–8.

31. Jaakkola T, Kalaja S, Liukkonen J, Jutila A, Virtanen P, Watt A. Relations among physical activity patterns, lifestyle activities, and fundamental movement skills for Finnish students in grade 7. *Percept Mot Skills* 2009;**108**:97–111.

32. Jago R, Baranowski T, Baranowski JC. Observed, GIS, and self-reported environmental features and adolescent physical activity. *Am J Health Promot* 2006;**20**:422–8.

33. Jago R, Baranowski T, Baranowski JC, Cullen KW, Thompson DI. Social desirability is associated with some physical activity, psychosocial variables and sedentary behavior but not self-reported physical activity among adolescent males. *Health Educ Res* 2007;**22**:438–49.

34. Janz KF, Mahoney LT. Maturation, gender, and video game playing are related to physical activity intensity in adolescents: The muscatine study. *Pediatr Exerc Sci* 1997;**9**:353–63.

35. Nitzan Kaluski D, Demem Mazengia G, Shimony T, Goldsmith R, Berry EM. Prevalence and determinants of physical activity and lifestyle in relation to obesity among schoolchildren in Israel. *Public Health Nutr* 2009;**12**:774–82.

36. Karaca A, Caglar E, Bilgili N, Ayaz S. Screen time of adolescents in an economically developing country: the case of Turkey. *Ann Hum Biol* 2011;**38**:28–33.

37. Katzmarzyk PT, Malina RM. Contribution of organized sports participation to estimated daily energy expenditure in youth. *Pediatr Exerc Sci* 1998;**10**:378–86.

38. Kerner MS, Kurrant AB, Kalinski MI. Leisure-time physical activity, sedentary behavior, and fitness of high school girls. *Eur J Sport Sci* 2004;**4**:1–17.

39. Koezuka N, Koo M, Allison KR, et al. The relationship between sedentary activities and physical inactivity among adolescents: results from the Canadian Community Health Survey. *J Adolesc Health* 2006;**39**:515–22.

40. Lajunen H-R, Keski-Rahkonen A, Pulkkinen L, Rose RJ, Rissanen A, Kaprio J. Leisure activity patterns and their associations with overweight: a prospective study among adolescents. *J Adolesc* 2009;**32**:1089–103.

41. Landsberg B, Plachta-Danielzik S, Lange D, Johannsen M, Seiberl J, Müller MJ. Clustering of lifestyle factors and association with overweight in adolescents of the Kiel Obesity Prevention Study. *Public Health Nutr* 2010;13:1708–15.

42. Lasserre AM, Chiolero A, Cachat F, Paccaud F, Bovet P. Overweight in Swiss children and associations with children’s and parents' characteristics. Obesity 2007;**15**:2912–9.

43. Leatherdale ST, Wong SL. Modifiable characteristics associated with sedentary behaviours among youth. *Int J Pediatr Obes* 2008;**3**:93–101.

44. Leatherdale ST, Faulkner G, Arbour-Nicitopoulos K. School and student characteristics associated with screen-time sedentary behavior among students in grades 5-8, Ontario, Canada, 2007-2008. *Prev Chronic Dis* 2010;**7**:A128.

45. Lippo BRS, Silva IM da, Aca CRP, Lira PIC de, Silva GAP da, Motta MEFA. Determinants of physical inactivity among urban adolescents. *J Pediatr* 2010;**86**:520–4.

46. Lobelo F, Dowda M, Pfeiffer KA, Pate RR. Electronic Media Exposure and Its Association With Activity-Related Outcomes in Female Adolescents: Cross-Sectional and Longitudinal Analyses. *J Phys Act Health* 2009;**6**:137–43.

47. Lowry R, Wechsler H, Galuska DA, Fulton JE, Kann L. Television viewing and its associations with overweight, sedentary lifestyle, and insufficient consumption of fruits and vegetables among US high school students: differences by race, ethnicity, and gender. *J Sch Health* 2002;**72**:413–21.

48. Maher C, Olds TS, Eisenmann JC, Dollman J. Screen time is more strongly associated than physical activity with overweight and obesity in 9- to 16-year-old Australians. *Acta Paediatr* 2012;**101**:1170–4.

49. Mamun A a, O’Callaghan MJ, Williams G, Najman JM. Television watching from adolescence to adulthood and its association with BMI, waist circumference, waist-to-hip ratio and obesity: a longitudinal study. *Public Health Nutr* 2013;**16**:54–64.

50. Mandic S, Bengoechea EG, Stevens E, de la Barra SL, Skidmore P. Getting kids active by participating in sport and doing it more often: focusing on what matters. *Int J Behav Nutr Phys Act* 2012;**9**:86.

51. Marshall SJ, Biddle SJH, Sallis JF, McKenzie TL, Conway TL. Clustering of sedentary Behaviors and physical activity among youth: A cross-national study. *Pediatr Exerc Sci* 2002;**14**:401–17.

52. Martinez-Gomez D, Eisenmann JC, Healy GN, et al. Sedentary behaviors and emerging cardiometabolic biomarkers in adolescents. *J Pediatr* 2012;**160**:104–10.e2.

53. Martínez-Gómez D, Veiga OL, Gomez-Martinez S, Zapatera B, Calle ME, Marcos A. Behavioural correlates of active commuting to school in Spanish adolescents: the AFINOS (Physical Activity as a Preventive Measure Against Overweight, Obesity, Infections, Allergies, and Cardiovascular Disease Risk Factors in Adolescents) study. *Public Health Nutr* 2011;**14**:1779–86.

54. McGuire MT, Neumark-Sztainer DR, Story M. Correlates of time spent in physical activity and television viewing in a multi-racial sample of adolescents. *Pediatr Exerc Sci* 2002;**14**:75–86.

55. Melkevik O, Torsheim T, Iannotti RJ, Wold B. Is spending time in screen-based sedentary behaviors associated with less physical activity: a cross national investigation. *Int J Behav Nutr Phys Act* 2010;**7**:46.

56. Mota J, Santos MP, Ribeiro JC. Differences in Leisure-Time Activities According to Level of Physical Activity in Adolescents. *J Phys Act Health* 2008;**5**:286–93.

57. Motl RW, McAuley E, Birnbaum AS, Lytle LA. Naturally occurring changes in time spent watching television are inversely related to frequency of physical activity during early adolescence. *J Adolesc* 2006;**29**:19–32.

58. Norman GJ, Schmid B a, Sallis JF, Calfas KJ, Patrick K. Psychosocial and environmental correlates of adolescent sedentary behaviors. *Pediatrics* 2005;**116**:908–16.

59. Pate RR, Heath GW, Dowda M, Trost SG. Associations between physical activity and other health behaviors in a representative sample of US adolescents. *Am J Public Health* 1996;**86**:1577–81.

60. Raudsepp L, Neissaar I, Kull M. Longitudinal stability of sedentary behaviors and physical activity during early adolescence. *Pediatr Exerc Sci* 2008;**20**:251–62.

61. Rivera IR, Silva MAM da, Silva RDTA, Oliveira BAV de, Carvalho ACC. Physical inactivity, TV-watching hours and body composition in children and adolescents. *Arq Bras Cardiol* 2010;**95**:159–65.

62. Robinson TN, Hammer LD, Killen JD, et al. Does television viewing increase obesity and reduce physical activity? Cross-sectional and longitudinal analyses among adolescent girls. *Pediatrics* 1993;**91**:273–80.

63. Robinson TN, Killen JD. Ethnic and gender differences in the relationship between television viewing and obesity, physical activity, and dietary fat intake. *J Heal Educ* 1995;**26**:s91–s98.

64. Ruiz JR, Ortega FB, Castillo R, et al. Physical activity, fitness, weight status, and cognitive performance in adolescents. *J Pediatr* 2010;**157**:917–922.

65. Sandercock GRH, Ogunleye A, Voss C. Screen Time and Physical Activity in Youth: Thief of Time or Lifestyle Choice? *J Phys Act Health* 2012;**9**:977–84.

66. Schneider M, Dunton GF, Cooper DM. Media use and obesity in adolescent females. *Obesity* 2007;**15**:2328–35.

67. Serrano-Sanchez JA, Martí-Trujillo S, Lera-Navarro A, Dorado-García C, González-Henríquez JJ, Sanchís-Moysi J. Associations between screen time and physical activity among Spanish adolescents. *PLoS One* 2011;**6**:e24453.

68. Sirard JR, Pfeiffer KA, Dowda M, Pate RR. Race differences in activity, fitness, and BMI in female eighth graders categorized by sports participation status. *Pediatr Exerc Sci* 2008;**20**:198–210.

69. Sjolie AN. School journeys and leisure activities in rural and urban adolescents in Norway. *Health Promot Int* 2002;**17**:21–30.

70. Snoek HM, van Strien T, Janssens JMAM, Engels RCME. The effect of television viewing on adolescents’ snacking: individual differences explained by external, restrained and emotional eating. *J Adolesc Health* 2006;**39**:448–51.

71. Spink KS, Chad K, Muhajarine N, et al. Intrapersonal correlates of sufficiently active youth and adolescents. *Pediatr Exerc Sci* 2005;**17**:124–35.

72. Strauss RS, Rodzilsky D, Burack G, Colin M. Psychosocial correlates of physical activity in healthy children. *Arch Pediatr Adolesc Med* 2001;**155**:897–902.

73. Strong LL, Anderson CB, Miranda PY, et al. Gender differences in sociodemographic and behavioral influences of physical activity in Mexican-origin adolescents. *J Phys Act Health* 2011;**9**:829–39.

74. Tammelin T, Ekelund U, Remes J, Näyhä S. Physical activity and sedentary behaviors among Finnish youth. *Med Sci Sports Exerc* 2007;**39**:1067–74.

75. Taveras EM, Field AE, Berkey CS, et al. Longitudinal relationship between television viewing and leisure-time physical activity during adolescence. *Pediatrics* 2007;**119**:e314–9.

76. Taverno Ross SE, Dowda M, Beets MW, Pate RR. Physical activity behavior and related characteristics of highly active eighth-grade girls. *J Adolesc Health* 2013;**52**:745–51.

77. Trang NHHD, Hong TK, Dibley MJ, Sibbritt DW. Factors associated with physical inactivity in adolescents in Ho Chi Minh City, Vietnam. *Med Sci Sports Exerc* 2009;**41**:1374–83.

78. Ussher MH, Owen CG, Cook DG, Whincup PH. The relationship between physical activity, sedentary behaviour and psychological wellbeing among adolescents. *Soc Psychiatry Psychiatr Epidemiol* 2007;**42**:851–6.

79. Van den Bulck J, Hofman A. The television-to-exercise ratio is a predictor of overweight in adolescents: results from a prospective cohort study with a two year follow up. *Prev Med* 2009;**48**:368–71.

80. Van der Sluis ME, Lien N, et al. Longitudinal associations of energy balance-related behaviours and cross-sectional associations of clusters and body mass index in Norwegian adolescents. *Public Health Nutr* 2010;**13**:1716–21.

81. Vilhjalmsson R, Thorlindsson T. Factors related to physical activity: a study of adolescents. *Soc Sci Med* 1998;**47**:665–75.

82. Wang CKJ, Chia YHM, Quek JJ, Liu WC. Patterns of physical activity, sedentary behaviors, and psychological determinants of physical activity among Singaporean school children. *Int J Sport Exerc Psychol* 2006;**4**:227–49.

83. Webb OJ, Benjamin CC, Gammon C, McKee HC, Biddle SJH. Physical activity, sedentary behaviour and physical self-perceptions in adolescent girls: A mediation analysis. *Ment Health Phys Act* 2013;**6**:24–9.

84. Williams SL, Mummery WK. Links between adolescent physical activity, body mass index, and adolescent and parent characteristics. *Health Educ Behav* 2011;**38**:510–20.

85. Wolf AM, Gortmaker SL, Cheung L, Gray HM, Herzog DB, Colditz GA. Activity, inactivity, and obesity: racial, ethnic, and age differences among schoolgirls. *Am J Public Health* 1993;**83**:1625–7.

86. Zabinski MF, Norman GJ, Sallis JF, Calfas KJ, Patrick K. Patterns of sedentary behavior among adolescents. *Health Psychol* 2007;**26**:113–20.
